# Emerging role of artificial intelligence in cardiac electrophysiology

**DOI:** 10.1016/j.cvdhj.2022.09.001

**Published:** 2022-09-27

**Authors:** Rajesh Kabra, Sharat Israni, Bharat Vijay, Chaitanya Baru, Raghuveer Mendu, Mark Fellman, Arun Sridhar, Pamela Mason, Jim W. Cheung, Luigi DiBiase, Srijoy Mahapatra, Jerome Kalifa, Steven A. Lubitz, Peter A. Noseworthy, Rachita Navara, David D. McManus, Mitchell Cohen, Mina K. Chung, Natalia Trayanova, Rakesh Gopinathannair, Dhanunjaya Lakkireddy

**Affiliations:** ∗ Kansas City Heart Rhythm Institute, Kansas City, Kansas; † Bakar Computational Health Sciences Institute, University of California, San Francisco, California; ‡ AmberTag Inc, Milpitas, California; § San Diego Supercomputer Center, University of California, San Diego, San Diego, California; ‖ NeuCures Inc, Los Angeles, California; ¶ Fellman Device Group LLC, Rockville, Maryland; # University of Washington, Seattle, Washington; ∗∗ Department of Medicine, University of Virginia, Charlottesville, Virginia; †† Division of Cardiology, Department of Medicine, Weill Cornell Medicine, New York, New York; ‡‡ Albert Einstein College of Medicine at Montefiore Hospital, New York, New York; §§ Department of Medicine, University of Minnesota, Minneapolis, Minnesota; ‖‖ Department of Cardiology, Brown University, Providence, Rhode Island; ¶¶ Cardiac Arrhythmia Service, Massachusetts General Hospital, Boston, Massachusetts; ## Department of Cardiovascular Medicine, Mayo Clinic, Rochester, Minnesota; ∗∗∗ Division of Cardiac Electrophysiology, University of California, San Francisco, San Francisco, California; ††† Department of Medicine, University of Massachusetts Chan Medical School, Worcester, Massachusetts; ‡‡‡ Division of Pediatric Cardiology, INOVA Children’s Hospital, Fairfax, Virginia; §§§ Division of Cardiovascular Medicine, Cleveland Clinic, Cleveland, Ohio; ‖‖‖ Department of Biomedical Engineering and Alliance for Cardiovascular Diagnostic and Treatment Innovation, Johns Hopkins University, Baltimore, Maryland

**Keywords:** Artificial intelligence, Machine learning, Deep learning, Cardiac electrophysiology, Big data, Computational modeling

## Abstract

Artificial intelligence (AI) and machine learning (ML) have significantly impacted the field of cardiovascular medicine, especially cardiac electrophysiology (EP), on multiple fronts. The goal of this review is to familiarize readers with the field of AI and ML and their emerging role in EP. The current review is divided into 3 sections. In the first section, we discuss the definitions and basics of AI, ML, and big data. In the second section, we discuss their application to EP in the context of detection, prediction, and management of arrhythmias. Finally, we discuss the regulatory issues, challenges, and future directions of AI in EP.

**Key Findings**

• Artificial Intelligence and machine learning have significantly impacted the field of cardiac electrophysiology.
• Application of AI to EKG, data from wearables and smart devices can provide information beyond human capabilities for risk stratification, disease screening, and detection of noncardiac conditions.
• AI can potentially be used to streamline workflow around remote monitoring of implantable cardiac devices, predict ICD therapies and response to CRT.
• AI can be used to identify sites of successful ablation and predict response to ablative therapies.
• Personalized computation modelling provides an individualized non-invasive approach to determine targets of ablation in ventricular tachycardia and persistent AF and determine arrhythmia risk in patients with heart disease

## Introduction—Artificial intelligence, machine learning, and big data

**Figure 1:**
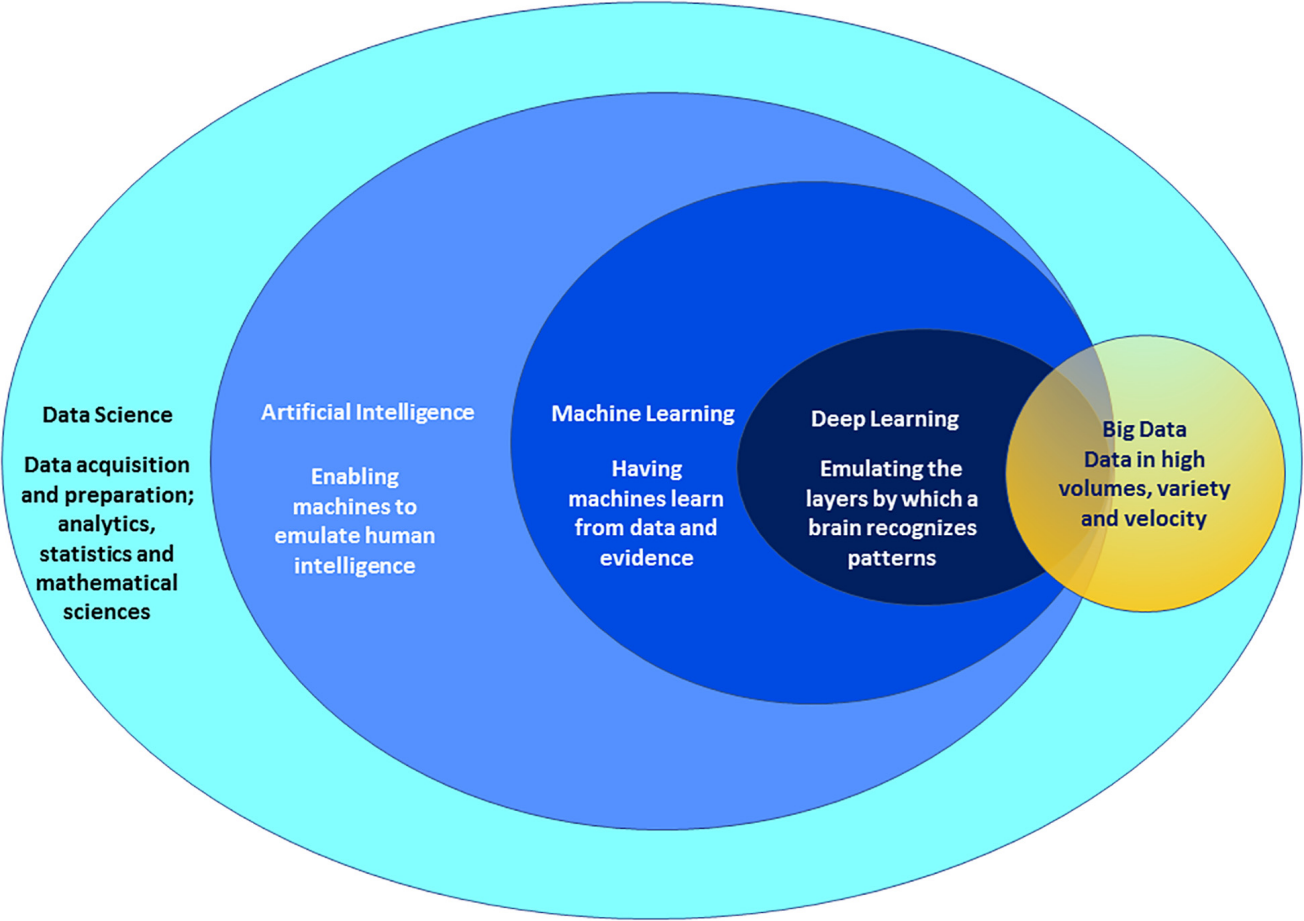
Positioning of disciplines commonly associated under the rubric of “AI.” This includes data science, artificial intelligence, machine learning, deep learning, and big data. Data velocity refers to the speed in which data are generated, distributed, and collected.

The 1956 Dartmouth Summer Research Project on Artificial Intelligence introduced the term “artificial intelligence” (AI) on the assumption that human intelligence “can be so precisely described that a machine can be made to simulate it.” Figure 1 shows the overview and definitions of the discipline of AI and its components.

The term “machine learning” (ML) was defined in 1959 by Arthur Samuel, who stated, “Programming computers to learn from experience should eventually eliminate the need for much of this detailed programming effort.” (https://ieeexplore.ieee.org/document/5392560). In ML, AI systems “learn” from the data that they are provided, complementing the original ideas of encoding “intelligence” into the AI system.

**Figure 2:**
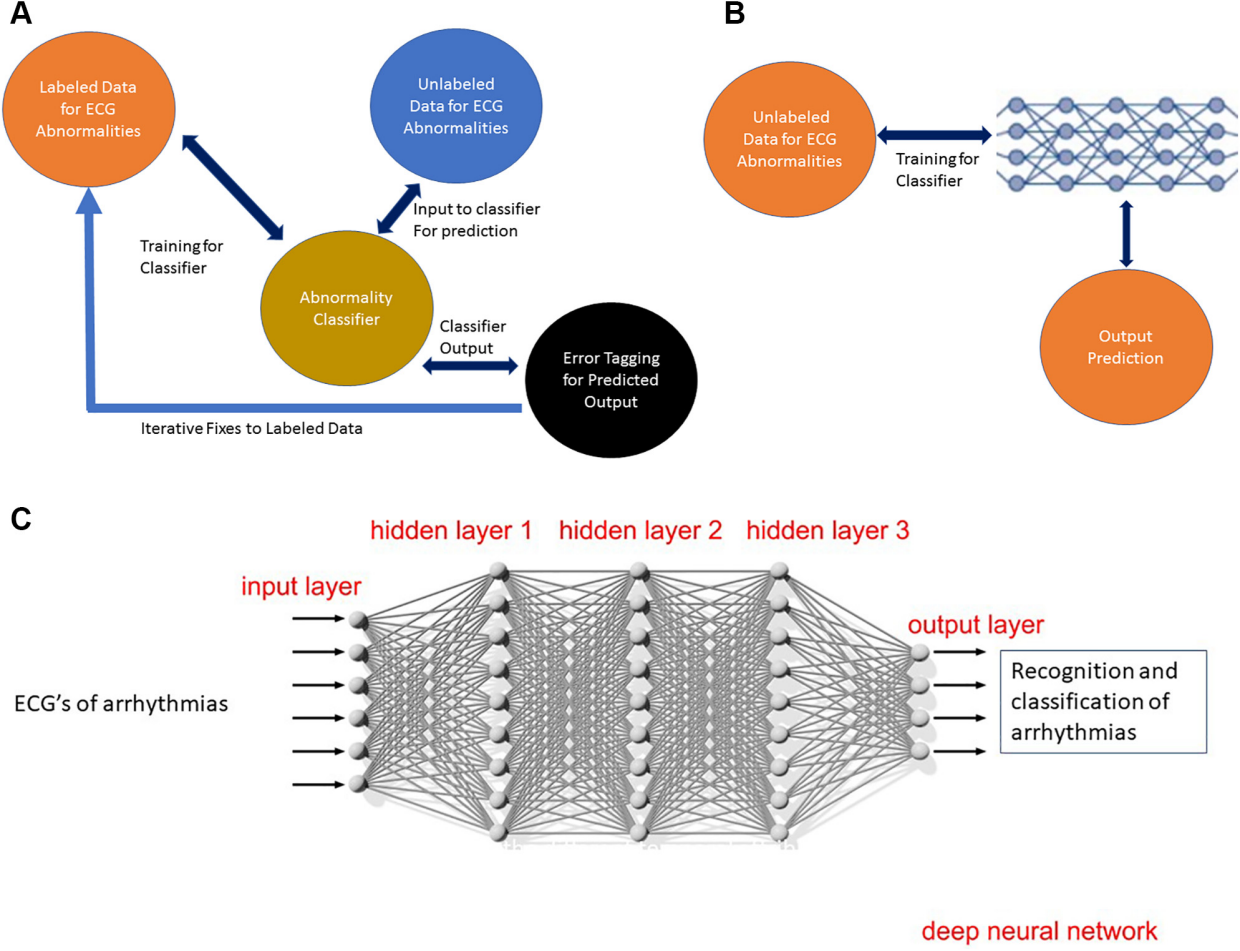
**A:** Supervised learning: The data for electrocardiogram (ECG) abnormality is labeled with human oversight and then used to train a classifier. Unlabeled data can then be classified by the classifier and the errors in classification can be measured with human oversight. Additional labeled data may be added to augment the Training Data to reduce error. An example would be classification of an abnormal rhythm (atrial fibrillation) from wearable devices using photoplethysmography in real time, after having been trained on labeled simulated signals. **B:** Unsupervised learning: The data for ECG is not labeled; many layers of the deep learning system iteratively learn the features and keep iteratively reducing the error with each run. **C:** Deep learning: ECGs of arrhythmias go through many layers of the deep learning system, which iteratively learns the features of arrhythmias and keeps reducing the error with each run to recognize and classify arrhythmias appropriately.

The ML process may be “supervised” or “unsupervised” (Figure 2A and 2B). In *supervised learning*, the machine is provided a set of labeled images as a source of truth, for instance electrocardiograms (ECG) labeled as sinus rhythm or atrial fibrillation (AF). For this approach to succeed, the right number of image features need to be identified, to accurately distinguish between right and wrong answers while not overfitting the model (being too specific) to this image set. This task of *feature extraction* can be prone to incompleteness or error. In contrast, *unsupervised learning* methods build the feature set by themselves, surfacing whatever they find of significance. For instance, the machine model may organize itself to find clinical factors in patient history that lead to a greater propensity for sudden cardiac death (SCD). These processes can be further refined by *reinforcement learning*, where the machine interacts with the environment and attempts to find an optimal way to achieve a desired goal. For instance, a drug treatment regimen model interacts with the “environment” (patient sub-phenotypes based on prior treatment regimens) to optimize treatments for individual patients. The efficiency of this model is improved by *dimensionality reduction,* which reduces the number of input factors to the most significant set in predicting desired outcomes. For example, to predict risk of AF, the model may start with multiple clinical factors, and finally identify only a few factors that are most important. This can make the model more user friendly.

*Deep learning* (DL) employs *artificial neural networks* inspired by the structure and functioning of the human brain. It is especially useful where the data are “unstructured”—such as images and text. Each layer (effectively, a step in a series) in the network performs a task, which is propagated to the next layer in the network (Figure 2C). There can be feedback to each layer from its downstream layer, to make adjustments. Iterations continue until the output has reached an acceptable level of accuracy. The number of processing layers through which data must pass is what inspired the term DL. For example, DL may be used to train AI models to recognize cardiac chambers or areas of abnormal cardiac tissue like myocardial scar or fibrosis from cardiac magnetic resonance imaging (MRI). Both ML and DL techniques depend on ingesting large amounts of data and employing statistical and predictive modeling techniques. A basic tenet of AI models is that even within 1 form of modeling, there may be many methods available. A collaboration with data scientists may help select the most appropriate model for the existing setting (eg, rare conditions with sparse data, or complex conditions or time to diagnosis in underserved communities). They may potentially even combine predictions from several models into 1 prediction using specialized techniques.

Cardiac electrophysiology (EP) presents a unique opportunity where device industry has a vast amount of patient data. Clinical providers are now forming partnerships that allow such data to be combined with the rest of the clinical records. Analyzing such big data in innovative ways can potentially provide insights and help make superior predictions about the behavior of complex systems, such as progression of AF or risk of SCD in a patient. While employing this general AI methodology, it is important to ensure that the data themselves are representative of the patient population where the AI models are to be deployed.

### AI framework in EP

**Figure 3:**
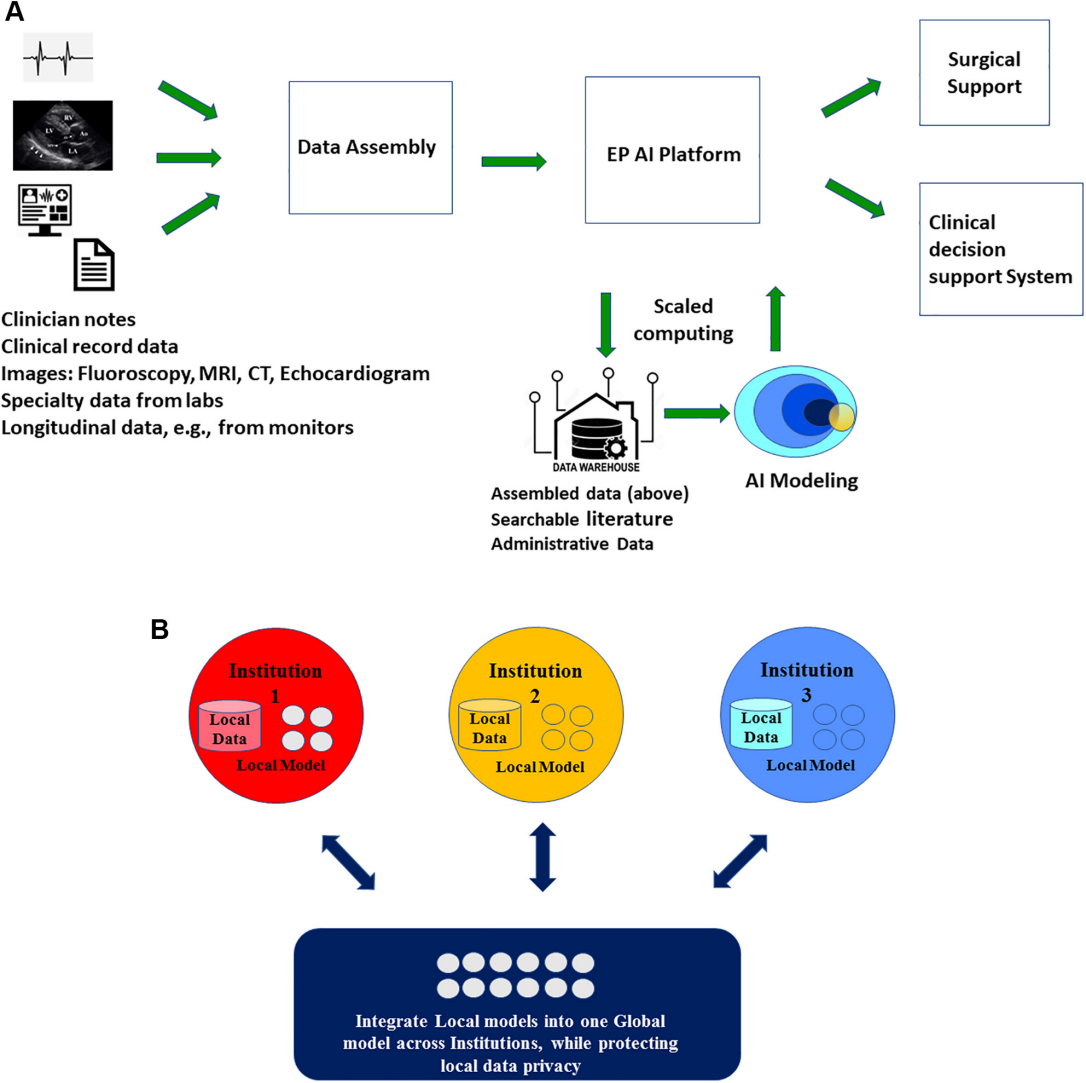
**A:** An artificial intelligence (AI) framework for clinical decision support in electrophysiology. Data from multiple sources are assembled, annotated, and processed by advanced computing and AI platform to generate models for clinical and surgical/procedural decision support. **B:** Federated data and modeling. In order to overcome restrictions on sharing of patients’ private data between institutions, models learn in a distributed fashion from data that are distributed and shared in a restrictive way.

AI and ML have a huge potential in the field of cardiac EP, where current patient data including ECG; information from wearables, smart devices, and implantable devices; and cardiac imaging may improve our understanding of mechanisms, risk prediction, and personalized treatment for arrhythmias like AF.^1^ Advanced imaging and mapping techniques can be used to accurately identify ablation targets and improve invasive and noninvasive treatment of arrhythmias. Figure 3A shows an AI framework for clinical decision support in EP. Multifactor data, with suitable computing and AI support, provide models for clinical and surgical decision support. The assembled data need human annotations to be AI-ready, especially in medical applications, where supervised learning dominates. Over time and with more data, the machine learns and classifies data patterns much better with the annotations.

A critical challenge in an emerging AI domain like EP is that only a few institutions have systematically collected sufficient data to create rich, robust models for clinical EP applications. Traditionally, such data have not been shared among centers owing to HIPAA compliance and preserving patient privacy. This creates an opportunity for EP to establish data-sharing standards and build relationships to create a new future for AI-enabled clinical medicine. AI models are much more difficult to create, and less impactful, when they are built on multiple small islands of data that are not co-located. Federated learning has emerged as an alternative where models learn from data that are distributed and shared in a restrictive way, so that patient privacy is maintained (Figure 3B).^2^

### AI and ECG

**Figure 4:**
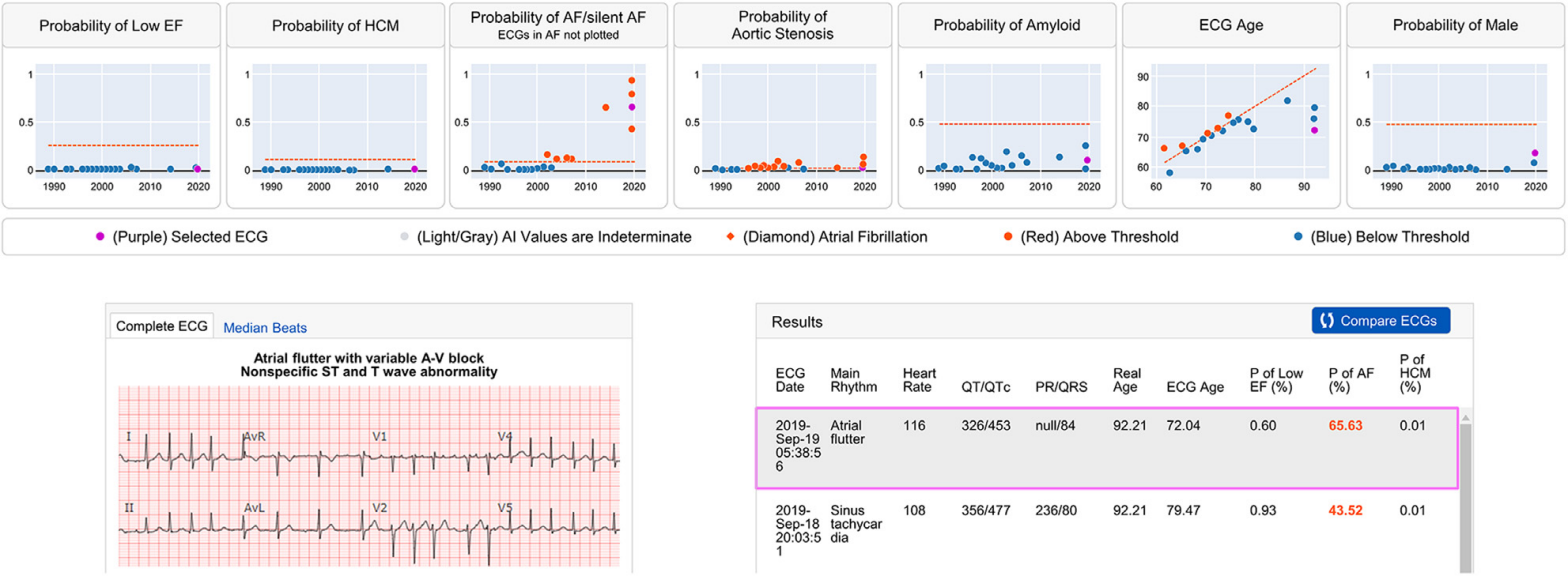
AI-ECG dashboard. All available electrocardiograms (ECGs) are analyzed in real time by a suite of algorithms and the results are displayed in a graphical and tabular format. In this example, the AI dashboard displays the output for models of low ejection fraction (EF), hypertrophic cardiomyopathy (HCM), silent atrial fibrillation (AF), aortic stenosis, cardiac amyloidosis, age, and sex. Each graph is laid out with time across the x-axis and model output (as a continuous variable from 0 to 1) along the y-axis. The threshold for a positive result (binary model threshold) is shown as a red line. All ECGs with model outputs below this threshold are shown in gray, whereas all positive results are shown in red. The ECG that is displayed as a 12-lead tracing (*highlighted*) is shown as a purple dot within each of the graphs.^11^, ^12^, ^13^, ^14^, ^15^, ^16^, ^17^, ^18^, ^19^

Although the ECG itself is nearly a century old, advances in computing power and the availability of digital ECG data with clinical linkage have fostered a renaissance for this humble diagnostic test. In general, the application of AI to the ECG can be seen as serving 2 main goals: (1) to create workflow efficiencies by performing *human-like* tasks in ECG interpretation (standard ECG annotation), or (2) to add value to the ECG through *extending human capability* by using the physiologic signal for risk stratification, disease screening, or detection of noncardiac conditions. Toward the former goal, the AI “sees” discernible patterns on the ECG in order to help cardiologists perform routine tasks; in the latter, the AI “sees beyond” human capability. The capacity for AI to perform human-like ECG interpretation has been demonstrated by several groups on single-lead^3^^,^^4^ and 12-lead ECG interpretation,^5^, ^6^, ^7^, ^8^ at times outperforming standard analog software.^9^ The ability to “see beyond” the human eye has been demonstrated by models that can detect patient sex or age,^10^ or even detect diseases that may not have a pathognomonic ECG signature, such as cardiac amyloidosis,^11^ pulmonary hypertension,^12^ aortic stenosis,^13^ hypertrophic cardiomyopathy,^14^ AF risk,^15^ concealed long QT syndrome,^16^ COVID-19,^17^ or low ejection fraction.^18^ Some of these models have been tested in prospective trials,^19^^,^^20^ while others are in earlier stages of development. As the clinical utility of these models becomes better established, next phases include validating them across multicenter datasets and integrating them into systems to make them available at the point of care. These models may be integrated into an “AI-ECG dashboard” that is linked from within the system’s electronic health record. This dashboard allows real-time AI analysis of all existing ECG data (Figure 4).

### AI and arrhythmia detection/prediction with wearables and smart devices

Technological innovations in mobile technology, including the production of wearables and smart devices, have created opportunities to measure physiologic data relevant to heart rhythm health. The widespread access to and consumer appeal of these devices has created a plethora of available data broadly accessible to the healthcare enterprise, including optical sensor–derived photoplethysmography (PPG) signals, ECGs, and accelerometers that enable measurement of physical activity. AI has potentially several clinical uses when applied to physiologic data from wearables or smart devices—classification of physiological data into clinically interpretable information, identification of disease states and prediction of future diseases, and clinical decision support. Examples of classification of physiologic data include the analysis of pulse waveform data from PPG that can infer the presence of an irregular heart rhythm,^21^ which has been studied most extensively for AF to date.^22^ Software operating on devices can further enable linkage of individuals to the healthcare enterprise. Algorithms coupled with PPG and pedometer data have been validated in a few studies for detecting AF.^23^^,^^24^ Newer active contact-free facial and fingertip plethysmography on smartphone cameras have shown potential for AF screening and diagnosis.^25^

AI algorithms applied to ECG data exhibit high discrimination for cardiac rhythms, including those measured using single-lead ECG patch monitors^26^ or mobile cardiac telemetry.^27^ Translating these exciting discoveries into improvements in clinical outcomes requires rigorous validation of algorithms, integration of data with the healthcare system, augmentation of existing clinical workflows, and robust access by patients. For consumer technology in particular, some aspects remain challenging, including ensuring that patients can effectively communicate results with providers^28^ as well as providing healthcare providers with formal guidance on the clinical use of mobile technology results with clinical care. AI incorporation has reached new horizons with the start of the COVID-19 pandemic. Recent innovations such as the TeleCheck-AF project, home antiarrhythmic drug loading with smartphone tracings, and smartphone ECG surveillance are a few great examples.^29^, ^30^, ^31^

AI algorithms that are applied to individual tracings, obtained from an ECG or PPG, for instance, examine only a small fraction of the available clinical data. It is likely that with time, broader models will be developed to aggregate data from multiple sensors as well as structured and unstructured data from the electronic health record, or other sources. In a recent study, AF prediction was significantly improved with the combination of clinical risk factors and AI-based ECG analysis.^32^

### AI in the management of cardiac implantable devices

Pacemakers and implantable cardioverter-defibrillators (ICDs) contain a wealth of therapeutic and diagnostic features. In particular, cardiac resynchronization therapy (CRT) devices and ICDs track variables that are known to correlate with worsening heart failure. Further, many patients with these devices are at risk for atrial and ventricular arrhythmias. The ability to predict and manage arrhythmias and heart failure events has significant implications for patients’ care, and the volume and complexity of the data makes it a prime area to benefit from AI.

In a successful risk factor model, 37 electrogram-based parameters from ICD data were successfully used to predict the risk of electrical storm.^33^ Although this is useful for only short-term prediction of ventricular tachycardia (VT) storm events, it is an important example of how the high-value data cardiovascular implantable electronic devices generate can be harnessed by ML, even in the absence of clinical parameters.

Remote monitoring has been shown to be as effective as in-clinic device checks for patients with pacemakers and ICDs and has shown to reduce shocks, facilitate early detection of device malfunction, and improve mortality.^34^ It is challenging for programs to manage the large volume of data that these systems generate. In addition to the high burden and quality of the data, different manufacturers have distinct algorithms and institutions have various mechanisms for collecting and managing these data, which makes this another important area to benefit from AI. Rosier and colleagues^35^ were able to show that a system that integrated patient’s medical history with pacemaker alert notifications was able to reduce the notification workload by 84%. Another study demonstrated that application of a 2-part AI filter to implantable loop recorder AF alerts was able to reduce the false-positive rate by more than 60%.^36^ Work is ongoing to devise ways to use AI to manage heart failure alerts more effectively.^37^

There has been considerable work to create ML models to predict response to CRT. Using the data from the major CRT trials, successful supervised learning and unsupervised learning models have been created.^38^^,^^39^ While many of these are complex to employ, a naïve Bayesian classifier that predicts CRT response using 9 variables led to the design of publicly available calculator and can be used in shared decision-making.^40^^,^^41^

### AI in ablation and mapping

During an ablation procedure, there is an exceptionally high information input: patient history, previous recordings, real-time navigation, electrograms, ablation parameters, ablation impact, and clinical follow-up. Historical description of intracardiac electrograms has been mono- or pauci-parametric: voltage amplitude, frequency, number of deflections, visually recognizable morphologies, or sequences. There is significant interobserver variation in the interpretation and significance of individual electrograms. While good targets are nearly impossible to describe with mono-parametric linear models, the ML/DL multiparametric nonlinear models are ideally suited to find outcome-relevant targets and treat patients. Multicenter randomized clinical trials are needed to demonstrate the usefulness of such analytical tools on the long-term outcomes of arrhythmia ablation. For example, one study is testing the hypothesis that the catheter ablation (CA) of ML/DL-adjudicated electrogram locations in addition to pulmonary vein isolation (PVI) is superior to PVI alone for patients in persistent AF (Tailored AF, NCT04702451).

In order to develop such AI capabilities for mapping and ablation, it is important for centers to pool data and help train expert systems using information about the patient (age, sex, race, and comorbidities), centers (multiple practices and operators), and equipment (mapping systems, recording systems, and catheters).

AI and ML can establish outcome prediction models for arrhythmia interventions such as AF CA.^42^ Recently, AI was used to study the prognostic potential of left atrial wall stress in AF recurrence after CA.^43^ A DL model using preablation pulmonary vein computed tomography predicted AF trigger origins with good reliability.^44^ Beyond atrial arrhythmias, ML has been applied to the detection and mapping of ventricular arrhythmias and to predicting the outcomes of cardiovascular implantable electronic device therapies. ML algorithms were shown to have excellent sensitivity and specificity for premature ventricular contraction detection and reliable localization of VT.^45^^,^^46^ Prediction of complications of arrhythmias can also benefit from AI owing to its ability to establish 3-dimensional structural models such as the left atrial appendage.^47^ A statistical *shape analysis* can provide further quantitative assessment of left atrial appendage shape and its association with stroke risk in AF patients.^48^ Lastly, novel intracardiac mapping modules incorporating ML were also reported in recent years to accurately reconstruct left atrial and pulmonary vein anatomy during AF ablation.^49^

### Synergistic application of ML and personalized computational modeling in arrhythmias and EP

The application of ML in arrhythmia and EP requires typically large datasets curated for specific applications. There are several practical obstacles to collecting and making medical data available to the AI community, including legal and ethical issues. Personalized computational modeling offers an attractive alternative to overcome these challenges and limitations. In contrast to traditional ML, in this form of AI, computer systems construct a personalized virtual-heart model (also referred to as a digital twin of the patient’s heart) that is unique to the patient and requires only 1 clinical scan. This model is used to assess the arrhythmogenic propensity of the patient’s disease-remodeled substrate. It is typically based on cardiac imaging that visualizes the individual geometry and disease remodeling of the heart, such as the distribution of scar and fibrosis in the ventricles and atria. Patient data can be combined with the results of computational modeling of patients’ arrhythmogenic propensity, as such results, when generated by personalized computational models of patients’ hearts, can be inexpensive to obtain. Furthermore, including results of computational modeling imparts mechanistic underpinning to the resulting AI model. As an application, computational modeling has been used for noninvasive prediction of the targets of ablation of VT and persistent AF,^50^^,^^51^ and for assessing arrhythmia risk in patients with ischemic cardiomyopathy^52^ and repaired tetralogy of Fallot.^53^

**Figure 5:**
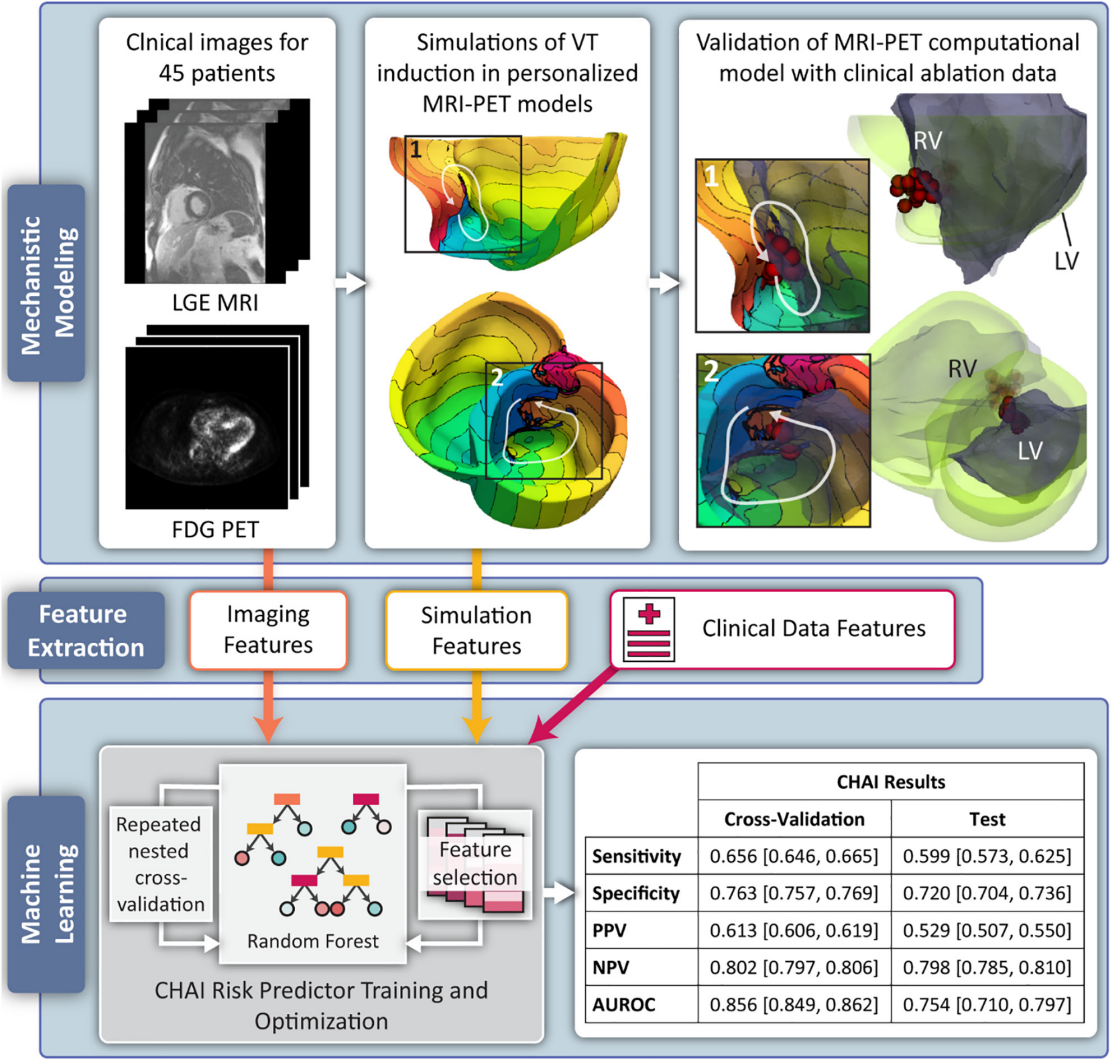
Overview of Computational Heart and Artificial Intelligence (CHAI) Risk Predictor Study. **Top panel, left:** Magnetic resonance imaging–positron emission tomography (MRI-PET) fusion models were created for 45 patients. Each model incorporates patient-specific ventricular geometry and spatial distributions of fibrosis and inflammation. **Top panel, middle:** Simulations of ventricular tachycardia (VT) induction were performed in each model. Isochrone lines represent activation times; line spacing is 22.5 ms. White arrows indicate reentry pathways. **Top panel, right:** Electroanatomic geometry (*gray*) is registered to computational model geometry (*green*) with clinical ablation lesions (*red circles*); insets show reentry pathways and ablation lesions. **Bottom panel, left:** Features from imaging, simulations, and clinical data are used as input to a supervised random forest classifier to predict risk of clinical VT. **Bottom panel, right:** Cross-validation and test results are aggregated over 560 iterations of nested cross-validation. Results are presented as mean [95% confidence interval]. AUROC = area under the receiver operation characteristic curve; FDG PET = 18[F]-fluorodeoxyglucose positron emission tomography; LGE-MRI = late gadolinium-enhanced magnetic resonance imaging; LV = left ventricle; NPV = negative predictive value; PPV = positive predictive value; RV = right ventricle.

Mechanistic computational modeling of the heart in an AI model presents novel precision technologies to predict risk of ventricular arrhythmias in patients with cardiac sarcoidosis^54^ and risk of AF recurrence following PVI in patients with paroxysmal AF.^55^ A supervised multivariable classifier learned both from the results of mechanistic modeling and from clinical and imaging biomarkers to predict clinical outcome. For instance, in the cardiac sarcoid study by Shade and colleagues,^54^ the mechanistic computational model was a novel MRI–positron emission tomography fusion model that assessed the arrhythmogenic propensity of the remodeled substrate incorporating fibrosis infiltration and inflammation. The combination with ML resulted in the creation of the Computational Heart and Artificial Intelligence (CHAI) Risk Predictor for patients with cardiac sarcoidosis (Figure 5). Personalized heart models of arrhythmogenesis were constructed using late gadolinium-enhanced MRI and positron emission tomography scans of the patient, and validated with clinical ablation data. Once the model was validated, features extracted from simulation results were combined with those extracted from imaging and other clinical data and fed into CHAI. In a cohort of 45 patients, the predictor achieved a balanced sensitivity and specificity and an excellent area under the receiver operating characteristic curve. In this proof-of-concept study, the CHAI results demonstrate that the technology outperformed clinical metrics in predicting risk of SCD in these patients.

A similar approach was used in a study predicting preprocedurally the risk of AF recurrence post-PVI in paroxysmal AF patients,^55^ where personalized atrial models were constructed from late gadolinium-enhanced MRI scans, and results of simulations of AF inducibility were used in a classifier together with other clinical data. These recent studies illustrate how the 2 approaches can be used synergistically and to overcome concerns over clinical decisions being informed by “black-box” algorithms that lack explainability. Such technologies pave the way for the use of integrative approaches in precision medicine that predict adverse events in complex diseases with a high degree of accuracy and mechanistic underpinning.

### AI in precision medicine

Next-generation sequencing technologies have greatly increased the availability of whole genome sequencing and high-resolution array genotyping and led to improvements in our understanding of the genetic basis for cardiovascular and arrhythmia disorders. The fulfillment of the promise of precision medicine to deliver patient-specific treatment for arrhythmias requires the synthesis and interpretation of data from multiple sources, including medical history, lifestyle and mobile health data, and rhythm monitoring data, as well as genomics, proteomics and metabolomics.^56^ The complexity and speed of this data accumulation makes it impractical for human-based analysis,^57^ particularly in the field of EP. ML is particularly suited to handle the tasks of integrating this wealth of clinical data with genetic data to inform disease classification, diagnosis, and treatment. Genomics, which seeks to correlate genotype and phenotype, identify biomarkers for patient stratification, and predict gene function, has become a dominant application for DL in healthcare.^58^

The range of potential applications of AI to drive precision medicine in EP is broad. For example, ML algorithms have been applied to identify abnormal calcium transient profiles in induced pluripotent stem cell–derived cardiomyocytes harboring mutations in genes associated with catecholaminergic polymorphic VT, long QT syndrome, and hypertrophic cardiomyopathy with classification accuracies of up to 87%.^59^ This could enhance efforts not only on genotype-phenotype correlation, but also on rapid examination of drug effects to guide treatment.^60^ Schmitz and colleagues^61^ examined the utility of 15 different ML algorithms that integrated both clinical and genetic parameters for the prediction of positive left ventricular remodeling with CRT. An optimal rule-based decision tree algorithm that incorporated clinical data and genotypes of genetic loci (eg, GNB3 rs5443 allele) predicted CRT response with >82.5% accuracy. Similar efforts to integrate genetic information to predict efficacy of antiarrhythmic drug therapy and CA procedures for arrhythmia therapy may be enhanced with ML. AF, which is often the end result of a complex interplay between genetic, clinical, and environmental factors, is one disease that may be best poised to be managed with the aid of ML-derived deep phenotyping and treatment optimization.

### AI and SCD prediction

It is well recognized that the current risk stratification models for SCD risk are very limited in determining ICD candidacy in patients with both structurally normal and abnormal hearts. There has been significant interest in developing better risk stratification models for risk of SCD using AI algorithms to harvest large population datasets.

**Figure 6:**
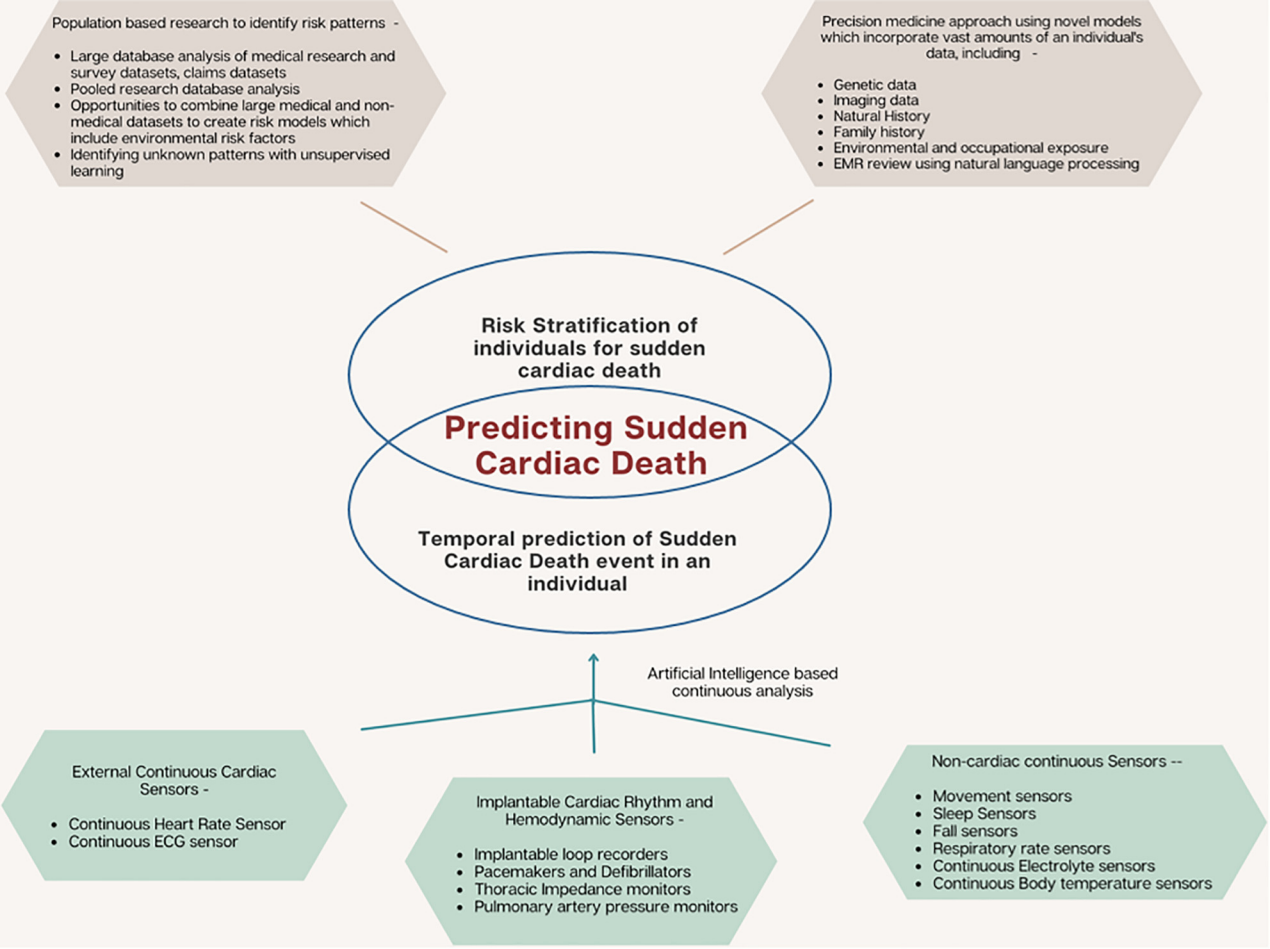
Artificial intelligence model for prediction of sudden cardiac death using multiple inputs. ECG = electrocardiogram; EMR = electronic medical records.

Various ML algorithms have been developed by different groups to predict the risk of SCD in individuals using clinical and demographic variables,^62^ ECG and Holter parameters,^63^ intracardiac electrogram characteristics derived from ICDs,^64^^,^^65^ imaging parameters from echocardiograms, MRIs and nuclear cardiac imaging,^66^ and even cellular phenotypes such as ventricular monophasic action potentials.^67^ The models use these parameters solely, or in different combinations as a multivariable classifier for SCD risk prediction,^68^ and have achieved variable successes with a wide range of areas under the curve (Figure 6). Apart from SCD risk prediction for individuals, there have also been attempts to use AI algorithms to temporally predict a higher risk of SCD in populations using complex meteorological and chronological data, showing that risk of SCD was higher with sudden temperature drops and on certain days of the week.^69^

Many of these algorithms serve as a proof of concept, demonstrating the capability of advanced computing and AI algorithms to derive patterns from extremely complex data. However, they are not close to clinical adoption at this time and require further prospective studies to pave the way for AI-guided risk stratification for SCD and determination of ICD candidacy.

## Regulatory framework and challenges for AI in EP

AI has the potential for significant impact on diagnosis and treatment of heart rhythm disorders and democratized care, but only if devices and software using AI can successfully navigate the US regulatory process. The Division of Digital Heath works interactively with the device-specific offices and stakeholders to discuss and develop policy and guidelines to support review of Software as a Medical Device (SaMD) submissions, including AI and ML.^70^^,^^71^ The US Food & Drug Administration’s (FDA) AI/ML SaMD Action Plan includes novel proposals like software precertification, predetermined change control, good ML practices, and real-world performance monitoring. Important FDA guidance has been issued for Clinical Decision Support software and Mobile Medical Applications.

Within the EP space, diagnostic software most commonly uses AI, and these products are primarily regulated as Class II SaMD devices. The pathway to market is either a 510(k) submission or de novo request.^72^^,^^73^ Developers may tune their Indications For Use, clinical utility claims, and patient population to optimize the regulatory process and level of evidence required to support a marketing application.^74^ Staged development strategies and presubmission interactions with the FDA are recommended.

Training and validation of data sources is a big challenge. The FDA expects studies to represent the US population, cover the intended patient group, have independent training/validation sources, and include prespecified analysis and endpoints. Solutions include the FDA’s National Evaluation System for Health Technology initiative,^75^ which is working to establish accessible data networks, including device registries, electronic health records, claims databases, and patient-generated health data. Coordination, cooperation, and linking of data sources by all EP SaMD stakeholders in an open and accessible network could accelerate the development and regulatory approval of EP SaMD incorporating AI.

### Limitations of AI in cardiac EP

Despite unparalleled advancement in AI across numerous healthcare domains, a number of limitations are present (Table 1). Large and diverse data sets with representation of normal and abnormal examples are required to increase both accuracy and generalizability of working models. The quality of the data is critical and the input data must reflect some level of heterogeneity to promote an iterative process. ML can also have errors or unexpected results. One of the more difficult concerns with AI in clinical EP is that algorithms can perform quite well during a test fit (training period) but when applied to the actual test set there is poor generalizability. This concept of “overfitting” within these very complex computational algorithms can be addressed by providing high-quality data at the time of training, careful monitoring of the training, and ensuring an optimal balance on the bias-variance trade-off curve.^76^ Furthermore, these complex computer results are mired by a certain level of opaqueness or black-box nature where it is challenging to understand how the result was generated.^77^ To achieve clinical usefulness and adoption of these algorithms for patient care, the development of explainable AI models, larger-scale multicenter controlled studies and thorough external validation of these algorithms are needed. Another limitation, especially in DL, relates to the challenge of integrating often complementary but separately obtained data on the same patient. How can MRI, nuclear testing, ECG, and imaging all be used in a large data set to create a functioning algorithm when the data points were obtained at varying time points? Large data sets should also bridge institutions, as the more complete and versatile data sets entered will establish better models. As always, AI should serve as an aid rather than a mandated voice to change clinical course. Clinicians should cautiously embrace the paradigm shift toward AI while striving to improve the computational models. No data set is valid and ready for AI without the right balance of data from diverse populations. It is well known that demographics, socioeconomics, genetics, and case histories can have an impact on the patient’s situation. The ethical collection and use of data are fundamental tenets of AI in medicine.

**Table 1:** Important challenges/obstacles to translating artificial intelligence to clinical practice and suggestions for overcoming them

Challenges and obstacles	Potential approaches
1. Lack of transparency (black box analyses) inhibits clinician uptake	Correlation analyses can sometimes help improve transparency. Use of new approaches, such as gradient-weighted class activation mapping, can help provide a level of interpretability.
2. Lack of validation and reproducibility in independent data sets	As the main reason behind this challenge is lack of access to independent data sets, any approaches that facilitate consistent data sharing would help alleviate the problem. For example, journals could require data be made public and provide a unified service that is HIPAA compliant and gives authorized users access.
3. Implementation in the EHR may be inhibited by regulatory requirements for clinical use	FDA review and advances may facilitate approval steps.
4. Need for strong technical teams, including data scientists, computer scientists, analysts; attracting skilled personnel to academics and medicine can be difficult, as industry offers higher salaries	Increase training pipelines. Provide institutional incentives for multidisciplinary approaches. Create and facilitate access to AI consulting services within each institution. Incorporate AI in medical training.
5. Need for large, harmonized quality data sets with representation of normal and abnormal examples and representation of data from diverse populations to avoid bias; HIPAA and need to preserve patient privacy can inhibit availability of large data sets for development	FDA National Evaluation System for health technology initiative – aims to establish accessible data networks, including device registries, EHR, claims databases, and patient-generated health data.

AI = artificial intelligence; EHR = electronic health records.

### Future directions of AI clinical electrophysiology

We stand at the precipice of a digital revolution that will forever change the landscape of EP. Although significant progress has been made in diagnostics and therapeutics, there are many gaps in our understanding of pathophysiology, risk stratification, and prevention. As outlined earlier in this manuscript, the medical field needs more collaboration in centralized global data collection, collation, and access to improve the accuracy and efficiency of many algorithms and processes. Upstream primary prevention and downstream secondary prevention models for disease management using genetic, molecular, demographic, clinical, and environmental data is an important directive for the future. This provides a real opportunity for basic scientists, clinicians, epidemiologists, computer scientists, and regulators to work together in creating robust infrastructure that facilitates open, accessible platforms for further creative development. Table 2 summarizes the future directions in the role of AI in EP.

**Table 2:** Future directions of artificial intelligence in electrophysiology and proposed studies to advance toward these future directions

Future directions	Approaches
1. Centralized global data collection, collation, and access to improve the accuracy and efficiency of many algorithms and processes	Greater collaboration and coordination among clinician scientists, professional societies, medical institutions, research organizations, and regulatory bodies, driven by governments and appropriate legislations to promote easy and open data exchange
2. Upstream primary prevention and downstream secondary prevention models for disease management using genetic, molecular, demographic, clinical, and environmental data	Consider focusing on individual diseases with a broader interlinking as one makes incremental progress in unlocking the pathophysiology and disease evolution
3. Creation of robust infrastructure that facilitates open, accessible platform	Interlinking of electronic medical records and epidemiological data with appropriate regulatory oversight

This infinite journey has to be mapped and staged in multiple progressive segments with continued appraisal of the precision, efficiency, and reliability of the prevailing AI/ML engines. Clinicians should consider focusing on individual diseases with a broader interlinking as one makes incremental progress in unlocking the secrets of pathophysiology and disease evolution. We live in a world of data silos. Interlinking electronic medical records and epidemiological data with appropriate regulatory oversight will be an important first step in the right direction.

## Conclusion

AI and ML have revolutionized the field of EP from detection and diagnosis of arrhythmia to risk prediction and management. Prediction of future diseases and outcomes based on current patient data including ECG, information from wearables/smart devices, and patient genomics offers a chance to provide personalized precision care to our patients. Advances in imaging, computational models for arrhythmia propensity, and accurate identification of ablation targets will improve invasive and noninvasive treatment of arrhythmias. Further advancement of this field is, however, dependent on availability of quality data, which may sometimes be challenging owing to HIPAA compliance and patient privacy. There are significant regulatory requirements that need to be navigated carefully. These are exciting times for the field of EP, where close collaboration between the clinicians, scientists, epidemiologists, and policy makers is the key to use technology for enhancing care of patients with cardiac arrhythmia.

## Funding Sources

### Disclosures

Rajesh Kabra – Fellowship support grants from Boston Scientific, Abbott, Medtronic, Biosense Webster; Sharat Israni – None; Bharat Vijay – Technology Leader, NeuTrace Inc; Chaitanya Baru: None; Raghuveer Mendu – Board member of NeuCures, NeuTrance, NeuFera, Orca systems, Seclore Inc; Rakesh Gopinathannair – Consultant/speaker: 10.13039/100000046Abbott Medical, 10.13039/100004319Pfizer, Boston Scientific, 10.13039/100007497Biosense Webster, Zoll Medical; Advisory Board (no compensation): Altathera, PaceMate; Mark Fellman – None; Arun Sridhar – None; Pamela 10.13039/100006369Mason – Consulting: 10.13039/100004374Medtronic and Boston Scientific; Honoraria; Cook; Jim W. Cheung – Consulting: Abbott, Boston Scientific, Biotronik; Fellowship grant support: Abbott, Boston Scientific, Biosense, 10.13039/501100005035Biotronik, and Medtronic; Luigi DiBiase – Consultant for 10.13039/100007497Biosense Webster, Stereotaxis, Rhythm management, 10.13039/100008497Boston Scientific, and Abbott; received speaker honoraria/travel from Medtronic, 10.13039/100004319Pfizer, 10.13039/501100005035Biotronik, and Baylis Medical; Srijoy Mahapatra – Shareholder of Neucures; Jerome Kalifa – Shareholder of Volta Medical; Steven A. Lubitz – Supported by NIH grant 1R01HL139731 and 10.13039/100000968American Heart Association 18SFRN34250007; Research support from Bristol Myers Squibb / 10.13039/100004319Pfizer, 10.13039/100004326Bayer AG, 10.13039/100001003Boehringer Ingelheim, Fitbit, and IBM, and has consulted for Bristol Myers Squibb / 10.13039/100004319Pfizer, 10.13039/100004326Bayer AG, and Blackstone Life Sciences; Peter A. Noseworthy – PAN and 10.13039/100000871Mayo Clinic have filed patents related to the application of AI to the ECG for diagnosis and risk stratification; PAN and 10.13039/100000871Mayo Clinic have licensed several AI-ECG algorithms to Anumana; PAN and 10.13039/100000871Mayo Clinic are involved in potential equity/royalty relationship with AliveCor related to an AI-ECG algorithm; study investigator in an ablation trial sponsored by Medtronic; served on an expert advisory panel for Optum; receives research funding from 10.13039/100000002National Institutes of Health (NIH, including the 10.13039/100000050National Heart, Lung, and Blood Institute [NHLBI, R21AG 62580-1, R01HL 131535-4, R01HL 143070-2] the 10.13039/100000049National Institute on Aging [NIA, R01AG 062436-1]), 10.13039/100000133Agency for Healthcare Research and Quality (AHRQ, R01HS 25402-3), 10.13039/100009210Food and Drug Administration (FDA, FD 06292), and the 10.13039/100000968American Heart Association (18SFRN34230146, AHA); Rachita Navara – Equity ownership in SafeBeat Rx; David D. McManus – supported by NIH grants U54HL143541, R01HL137734, R01HL155343, R01HL137794, R01HL141434, and R61HL158541 as primary or multi-primary investigator and grants and personal fees from Bristol Myers Squibb, grants and personal fees from Pfizer, grants from 10.13039/100001003Boehringer Ingelheim and 10.13039/100004320Philips, nonfinancial support from Apple, personal fees and nonfinancial support from 10.13039/100004358Samsung, grants and personal fees from Flexcon, personal fees from Avania, personal fees from Rose Consulting, grants and personal fees from Heart Rhythm Society, and personal fees and nonfinancial support from Fitbit; Mitchell Cohen – None; Mina Chung – NIH grant R01 HL158071; 10.13039/100000968American Heart Association Atrial Fibrillation Strategically Focused Research Network grants 18SFRN34110067, 18SFRN34170013, 20SCG35490449; Natalia Trayanova – NIH grants R01HL142496 and U01HL141074, a grant from the Leducq Foundation; Dhanunjaya Lakkireddy – Consulting: Abbott, Biosense Webster, Medtronic, Boston Scientific, 10.13039/501100005035Biotronik, 10.13039/100004331Johnson & Johnson, Acutus, BioTel, AltaThera, Northeast Scientific.

## Authorship

All authors attest they meet the current ICMJE criteria for authorship.
